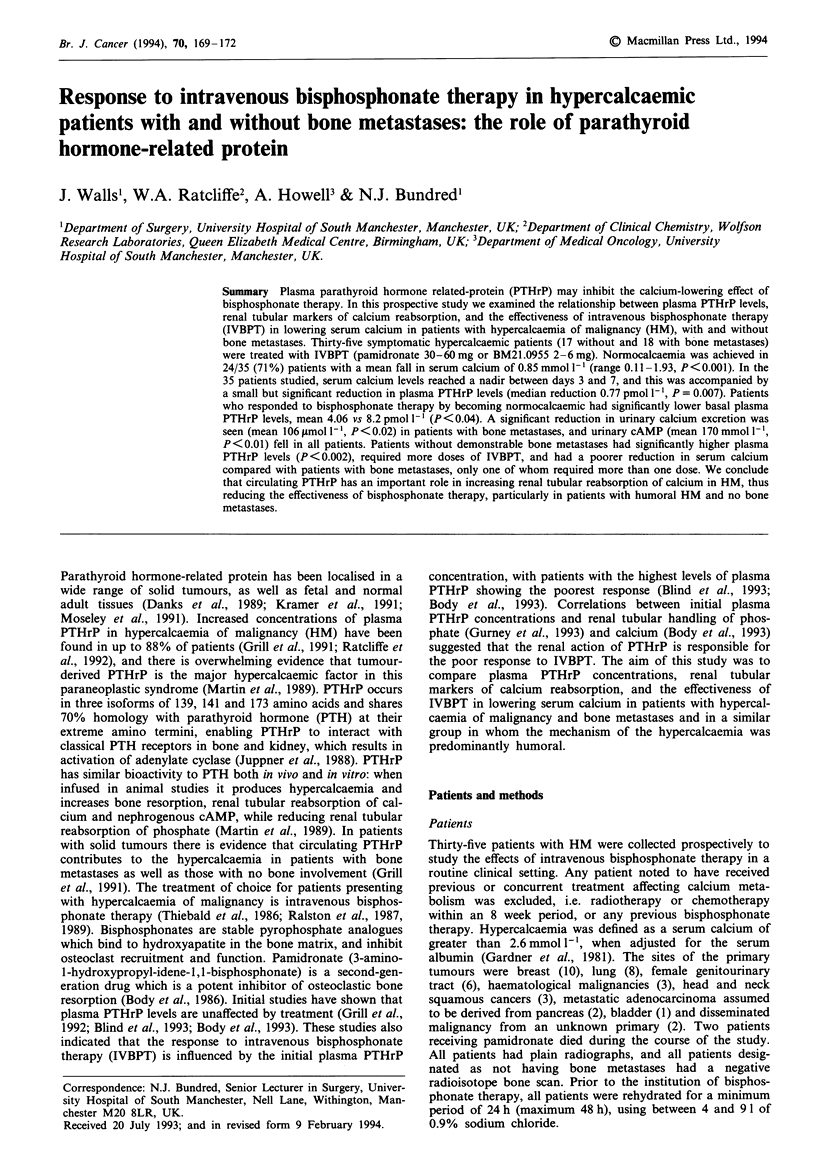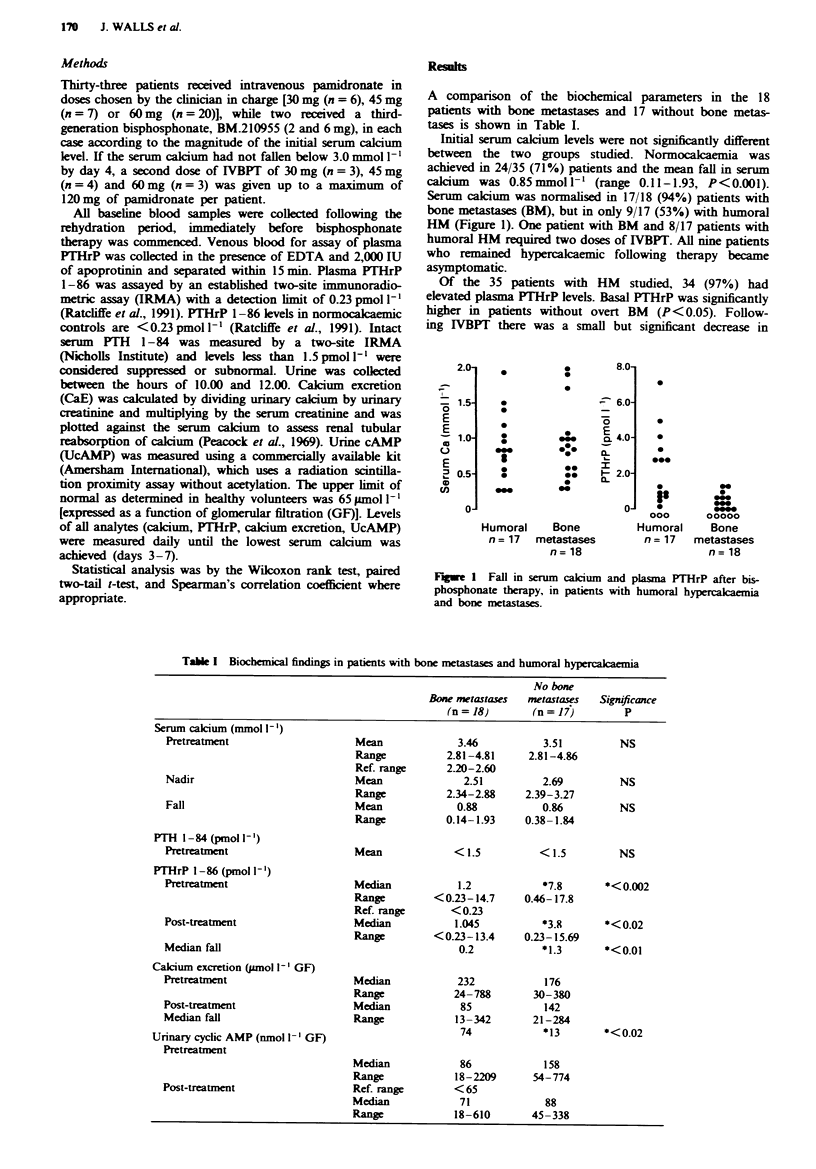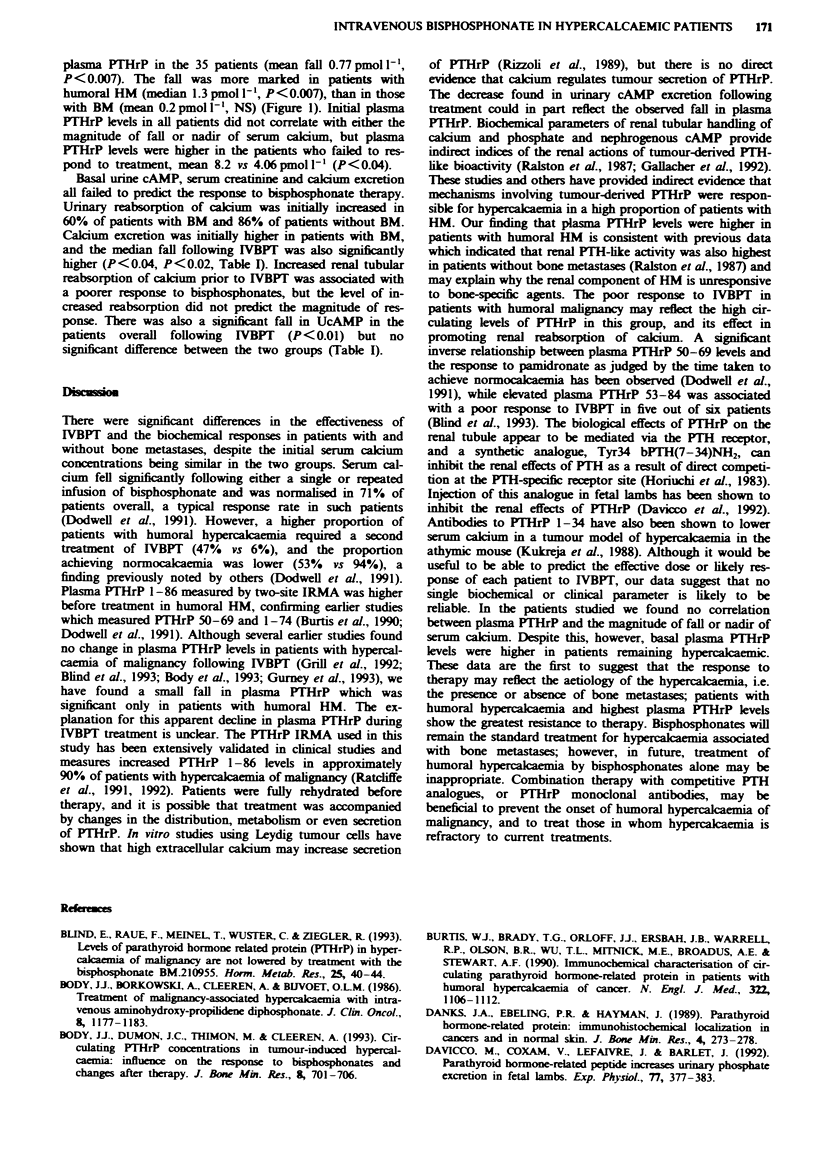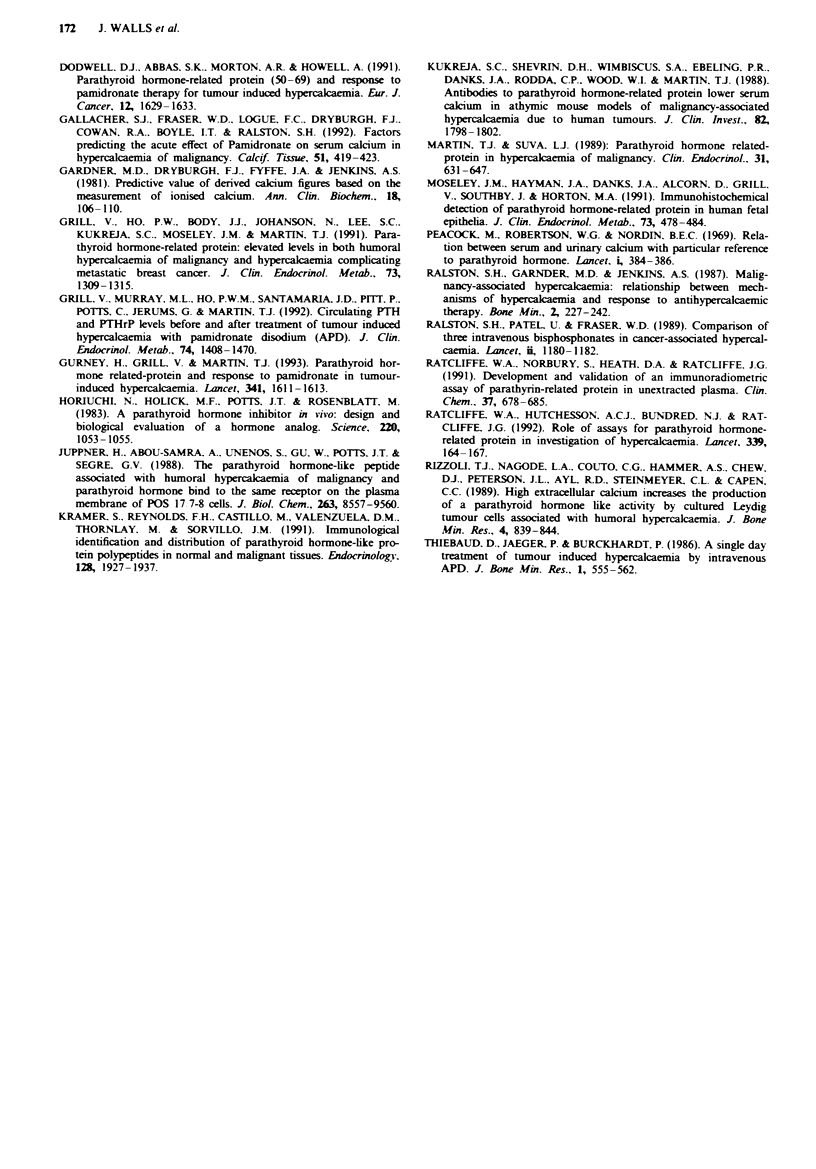# Response to intravenous bisphosphonate therapy in hypercalcaemic patients with and without bone metastases: the role of parathyroid hormone-related protein.

**DOI:** 10.1038/bjc.1994.270

**Published:** 1994-07

**Authors:** J. Walls, W. A. Ratcliffe, A. Howell, N. J. Bundred

**Affiliations:** Department of Surgery, University Hospital of South Manchester, UK.

## Abstract

Plasma parathyroid hormone related-protein (PTHrP) may inhibit the calcium-lowering effect of bisphosphonate therapy. In this prospective study we examined the relationship between plasma PTHrP levels, renal tubular markers of calcium reabsorption, and the effectiveness of intravenous bisphosphonate therapy (IVBPT) in lowering serum calcium in patients with hypercalcaemia of malignancy (HM), with and without bone metastases. Thirty-five symptomatic hypercalcaemic patients (17 without and 18 with bone metastases) were treated with IVBPT (pamidronate 30-60 mg or BM21.0955 2-6 mg). Normocalcaemia was achieved in 24/35 (71%) patients with a mean fall in serum calcium of 0.85 mmol l-1 (range 0.11-1.93, P < 0.001). In the 35 patients studied, serum calcium levels reached a nadir between days 3 and 7, and this was accompanied by a small but significant reduction in plasma PTHrP levels (median reduction 0.77 pmol l-1, P = 0.007). Patients who responded to bisphosphonate therapy by becoming normocalcaemic had significantly lower basal plasma PTHrP levels, mean 4.06 vs 8.2 pmol l-1 (P < 0.04). A significant reduction in urinary calcium excretion was seen (mean 106 mumol l-1, P < 0.02) in patients with bone metastases, and urinary cAMP (mean 170 mmol l-1, P < 0.01) fell in all patients. Patients without demonstrable bone metastases had significantly higher plasma PTHrP levels (P < 0.002), required more doses of IVBPT, and had a poorer reduction in serum calcium compared with patients with bone metastases, only one of whom required more than one dose. We conclude that circulating PTHrP has an important role in increasing renal tubular reabsorption of calcium in HM, thus reducing the effectiveness of bisphosphonate therapy, particularly in patients with humoral HM and no bone metastases.


*Br. J. Cancer* (1994), **70**, 169–172
© Macmillan Press Ltd., 1994

### Response to intravenous bisphosphonate therapy in hypercalcaemic patients with and without bone metastases: the role of parathyroid hormone-related protein

J. Walls[1], W.A. Ratcliffe[2], A. Howell[3] & N.J. Bundred[1]

[1]*Department of Surgery, University Hospital of South Manchester, Manchester, UK;* [2]*Department of Clinical Chemistry, Wolfson Research Laboratories, Queen Elizabeth Medical Centre, Birmingham, UK;* [3]*Department of Medical Oncology, University Hospital of South Manchester, Manchester, UK.*

**Summary** Plasma parathyroid hormone related-protein (PTHrP) may inhibit the calcium-lowering effect of bisphosphonate therapy. In this prospective study we examined the relationship between plasma PTHrP levels, renal tubular markers of calcium reabsorption, and the effectiveness of intravenous bisphosphonate therapy (IVBPT) in lowering serum calcium in patients with hypercalcaemia of malignancy (HM), with and without bone metastases. Thirty-five symptomatic hypercalcaemic patients (17 without and 18 with bone metastases) were treated with IVBPT (pamidronate 30–60 mg or BM21.0955 2–6 mg). Normocalcaemia was achieved in 24/35 (71%) patients with a mean fall in serum calcium of 0.85 mmol $l^{-1}$ (range 0.11–1.93, $P<0.001$). In the 35 patients studied, serum calcium levels reached a nadir between days 3 and 7, and this was accompanied by a small but significant reduction in plasma PTHrP levels (median reduction 0.77 pmol $l^{-1}$, $P=0.007$). Patients who responded to bisphosphonate therapy by becoming normocalcaemic had significantly lower basal plasma PTHrP levels, mean 4.06 *vs* 8.2 pmol $l^{-1}$ ($P<0.04$). A significant reduction in urinary calcium excretion was seen (mean 106 μmol $l^{-1}$, $P<0.02$) in patients with bone metastases, and urinary cAMP (mean 170 mmol $l^{-1}$, $P<0.01$) fell in all patients. Patients without demonstrable bone metastases had significantly higher plasma PTHrP levels ($P<0.002$), required more doses of IVBPT, and had a poorer reduction in serum calcium compared with patients with bone metastases, only one of whom required more than one dose. We conclude that circulating PTHrP has an important role in increasing renal tubular reabsorption of calcium in HM, thus reducing the effectiveness of bisphosphonate therapy, particularly in patients with humoral HM and no bone metastases.

Parathyroid hormone-related protein has been localised in a wide range of solid tumours, as well as fetal and normal adult tissues (Danks *et al.*, 1989; Kramer *et al.*, 1991; Moseley *et al.*, 1991). Increased concentrations of plasma PTHrP in hypercalcaemia of malignancy (HM) have been found in up to 88% of patients (Grill *et al.*, 1991; Ratcliffe *et al.*, 1992), and there is overwhelming evidence that tumour-derived PTHrP is the major hypercalcaemic factor in this paraneoplastic syndrome (Martin *et al.*, 1989). PTHrP occurs in three isoforms of 139, 141 and 173 amino acids and shares 70% homology with parathyroid hormone (PTH) at their extreme amino termini, enabling PTHrP to interact with classical PTH receptors in bone and kidney, which results in activation of adenylate cyclase (Juppner *et al.*, 1988). PTHrP has similar bioactivity to PTH both *in vivo* and *in vitro*: when infused in animal studies it produces hypercalcaemia and increases bone resorption, renal tubular reabsorption of calcium and nephrogenous cAMP, while reducing renal tubular reabsorption of phosphate (Martin *et al.*, 1989). In patients with solid tumours there is evidence that circulating PTHrP contributes to the hypercalcaemia in patients with bone metastases as well as those with no bone involvement (Grill *et al.*, 1991). The treatment of choice for patients presenting with hypercalcaemia of malignancy is intravenous bisphosphonate therapy (Thiebald *et al.*, 1986; Ralston *et al.*, 1987, 1989). Bisphosphonates are stable pyrophosphate analogues which bind to hydroxyapatite in the bone matrix, and inhibit osteoclast recruitment and function. Pamidronate (3-amino-1-hydroxypropyl-idene-1,1-bisphosphonate) is a second-generation drug which is a potent inhibitor of osteoclastic bone resorption (Body *et al.*, 1986). Initial studies have shown that plasma PTHrP levels are unaffected by treatment (Grill *et al.*, 1992; Blind *et al.*, 1993; Body *et al.*, 1993). These studies also indicated that the response to intravenous bisphosphonate therapy (IVBPT) is influenced by the initial plasma PTHrP concentration, with patients with the highest levels of plasma PTHrP showing the poorest response (Blind *et al.*, 1993; Body *et al.*, 1993). Correlations between initial plasma PTHrP concentrations and renal tubular handling of phosphate (Gurney *et al.*, 1993) and calcium (Body *et al.*, 1993) suggested that the renal action of PTHrP is responsible for the poor response to IVBPT. The aim of this study was to compare plasma PTHrP concentrations, renal tubular markers of calcium reabsorption, and the effectiveness of IVBPT in lowering serum calcium in patients with hypercalcaemia of malignancy and bone metastases and in a similar group in whom the mechanism of the hypercalcaemia was predominantly humoral.

### Patients and methods

### *Patients*

Thirty-five patients with HM were collected prospectively to study the effects of intravenous bisphosphonate therapy in a routine clinical setting. Any patient noted to have received previous or concurrent treatment affecting calcium metabolism was excluded, i.e. radiotherapy or chemotherapy within an 8 week period, or any previous bisphosphonate therapy. Hypercalcaemia was defined as a serum calcium of greater than 2.6 mmol $l^{-1}$, when adjusted for the serum albumin (Gardner *et al.*, 1981). The sites of the primary tumours were breast (10), lung (8), female genitourinary tract (6), haematological malignancies (3), head and neck squamous cancers (3), metastatic adenocarcinoma assumed to be derived from pancreas (2), bladder (1) and disseminated malignancy from an unknown primary (2). Two patients receiving pamidronate died during the course of the study. All patients had plain radiographs, and all patients designated as not having bone metastases had a negative radioisotope bone scan. Prior to the institution of bisphosphonate therapy, all patients were rehydrated for a minimum period of 24 h (maximum 48 h), using between 4 and 9 l of 0.9% sodium chloride.

Correspondence: N.J. Bundred, Senior Lecturer in Surgery, University Hospital of South Manchester, Nell Lane, Withington, Manchester M20 8LR, UK.
Received 20 July 1993; and in revised form 9 February 1994.

*Methods*

Thirty-three patients received intravenous pamidronate in doses chosen by the clinician in charge [30 mg ($n = 6$), 45 mg ($n = 7$) or 60 mg ($n = 20$)], while two received a third-generation bisphosphonate, BM.210955 (2 and 6 mg), in each case according to the magnitude of the initial serum calcium level. If the serum calcium had not fallen below 3.0 mmol $l^{-1}$ by day 4, a second dose of IVBPT of 30 mg ($n = 3$), 45 mg ($n = 4$) and 60 mg ($n = 3$) was given up to a maximum of 120 mg of pamidronate per patient.

All baseline blood samples were collected following the rehydration period, immediately before bisphosphonate therapy was commenced. Venous blood for assay of plasma PTHrP was collected in the presence of EDTA and 2,000 IU of apoprotinin and separated within 15 min. Plasma PTHrP 1-86 was assayed by an established two-site immunoradiometric assay (IRMA) with a detection limit of 0.23 pmol $l^{-1}$ (Ratcliffe *et al.*, 1991). PTHrP 1-86 levels in normocalcaemic controls are $<0.23$ pmol $l^{-1}$ (Ratcliffe *et al.*, 1991). Intact serum PTH 1-84 was measured by a two-site IRMA (Nicholls Institute) and levels less than 1.5 pmol $l^{-1}$ were considered suppressed or subnormal. Urine was collected between the hours of 10.00 and 12.00. Calcium excretion (CaE) was calculated by dividing urinary calcium by urinary creatinine and multiplying by the serum creatinine and was plotted against the serum calcium to assess renal tubular reabsorption of calcium (Peacock *et al.*, 1969). Urine cAMP (UcAMP) was measured using a commercially available kit (Amersham International), which uses a radiation scintillation proximity assay without acetylation. The upper limit of normal as determined in healthy volunteers was 65 μmol $l^{-1}$ [expressed as a function of glomerular filtration (GF)]. Levels of all analytes (calcium, PTHrP, calcium excretion, UcAMP) were measured daily until the lowest serum calcium was achieved (days 3-7).

Statistical analysis was by the Wilcoxon rank test, paired two-tail $t$-test, and Spearman's correlation coefficient where appropriate.

**Results**

A comparison of the biochemical parameters in the 18 patients with bone metastases and 17 without bone metastases is shown in Table I.

Initial serum calcium levels were not significantly different between the two groups studied. Normocalcaemia was achieved in 24/35 (71%) patients and the mean fall in serum calcium was 0.85 mmol $l^{-1}$ (range 0.11-1.93, $P<0.001$). Serum calcium was normalised in 17/18 (94%) patients with bone metastases (BM), but in only 9/17 (53%) with humoral HM (Figure 1). One patient with BM and 8/17 patients with humoral HM required two doses of IVBPT. All nine patients who remained hypercalcaemic following therapy became asymptomatic.

Of the 35 patients with HM studied, 34 (97%) had elevated plasma PTHrP levels. Basal PTHrP was significantly higher in patients without overt BM ($P<0.05$). Following IVBPT there was a small but significant decrease in

**Figure 1** Fall in serum calcium and plasma PTHrP after bisphosphonate therapy, in patients with humoral hypercalcaemia and bone metastases.

**Table I** Biochemical findings in patients with bone metastases and humoral hypercalcaemia

| | | *Bone metastases* (n = *18*) | *No bone metastases* (n = *17*) | *Significance* P |
|---|---|---|---|---|
| Serum calcium (mmol $l^{-1}$) | | | | |
| Pretreatment | Mean | 3.46 | 3.51 | NS |
| | Range | 2.81-4.81 | 2.81-4.86 | |
| | Ref. range | 2.20-2.60 | | |
| Nadir | Mean | 2.51 | 2.69 | NS |
| | Range | 2.34-2.88 | 2.39-3.27 | |
| Fall | Mean | 0.88 | 0.86 | NS |
| | Range | 0.14-1.93 | 0.38-1.84 | |
| PTH 1-84 (pmol $l^{-1}$) | | | | |
| Pretreatment | Mean | <1.5 | <1.5 | NS |
| PTHrP 1-86 (pmol $l^{-1}$) | | | | |
| Pretreatment | Median | 1.2 | *7.8 | *<0.002 |
| | Range | <0.23-14.7 | 0.46-17.8 | |
| | Ref. range | <0.23 | | |
| Post-treatment | Median | 1.045 | *3.8 | *<0.02 |
| | Range | <0.23-13.4 | 0.23-15.69 | |
| Median fall | | 0.2 | *1.3 | *<0.01 |
| Calcium excretion (μmol $l^{-1}$ GF) | | | | |
| Pretreatment | Median | 232 | 176 | |
| | Range | 24-788 | 30-380 | |
| Post-treatment | Median | 85 | 142 | |
| Median fall | Range | 13-342 | 21-284 | |
| Urinary cyclic AMP (nmol $l^{-1}$ GF) | | 74 | *13 | *<0.02 |
| Pretreatment | | | | |
| | Median | 86 | 158 | |
| | Range | 18-2209 | 54-774 | |
| Post-treatment | Ref. range | <65 | | |
| | Median | 71 | 88 | |
| | Range | 18-610 | 45-338 | |

plasma PTHrP in the 35 patients (mean fall 0.77 pmol $l^{-1}$, $P<0.007$). The fall was more marked in patients with humoral HM (median 1.3 pmol $l^{-1}$, $P<0.007$), than in those with BM (mean 0.2 pmol $l^{-1}$, NS) (Figure 1). Initial plasma PTHrP levels in all patients did not correlate with either the magnitude of fall or nadir of serum calcium, but plasma PTHrP levels were higher in the patients who failed to respond to treatment, mean 8.2 *vs* 4.06 pmol $l^{-1}$ ($P<0.04$).

Basal urine cAMP, serum creatinine and calcium excretion all failed to predict the response to bisphosphonate therapy. Urinary reabsorption of calcium was initially increased in 60% of patients with BM and 86% of patients without BM. Calcium excretion was initially higher in patients with BM, and the median fall following IVBPT was also significantly higher ($P<0.04$, $P<0.02$, Table I). Increased renal tubular reabsorption of calcium prior to IVBPT was associated with a poorer response to bisphosphonates, but the level of increased reabsorption did not predict the magnitude of response. There was also a significant fall in UcAMP in the patients overall following IVBPT ($P<0.01$) but no significant difference between the two groups (Table I).

### Discussion

There were significant differences in the effectiveness of IVBPT and the biochemical responses in patients with and without bone metastases, despite the initial serum calcium concentrations being similar in the two groups. Serum calcium fell significantly following either a single or repeated infusion of bisphosphonate and was normalised in 71% of patients overall, a typical response rate in such patients (Dodwell *et al.*, 1991). However, a higher proportion of patients with humoral hypercalcaemia required a second treatment of IVBPT (47% *vs* 6%), and the proportion achieving normocalcaemia was lower (53% *vs* 94%), a finding previously noted by others (Dodwell *et al.*, 1991). Plasma PTHrP 1-86 measured by two-site IRMA was higher before treatment in humoral HM, confirming earlier studies which measured PTHrP 50-69 and 1-74 (Burtis *et al.*, 1990; Dodwell *et al.*, 1991). Although several earlier studies found no change in plasma PTHrP levels in patients with hypercalcaemia of malignancy following IVBPT (Grill *et al.*, 1992; Blind *et al.*, 1993; Body *et al.*, 1993; Gurney *et al.*, 1993), we have found a small fall in plasma PTHrP which was significant only in patients with humoral HM. The explanation for this apparent decline in plasma PTHrP during IVBPT treatment is unclear. The PTHrP IRMA used in this study has been extensively validated in clinical studies and measures increased PTHrP 1-86 levels in approximately 90% of patients with hypercalcaemia of malignancy (Ratcliffe *et al.*, 1991, 1992). Patients were fully rehydrated before therapy, and it is possible that treatment was accompanied by changes in the distribution, metabolism or even secretion of PTHrP. *In vitro* studies using Leydig tumour cells have shown that high extracellular calcium may increase secretion of PTHrP (Rizzoli *et al.*, 1989), but there is no direct evidence that calcium regulates tumour secretion of PTHrP. The decrease found in urinary cAMP excretion following treatment could in part reflect the observed fall in plasma PTHrP. Biochemical parameters of renal tubular handling of calcium and phosphate and nephrogenous cAMP provide indirect indices of the renal actions of tumour-derived PTH-like bioactivity (Ralston *et al.*, 1987; Gallacher *et al.*, 1992). These studies and others have provided indirect evidence that mechanisms involving tumour-derived PTHrP were responsible for hypercalcaemia in a high proportion of patients with HM. Our finding that plasma PTHrP levels were higher in patients with humoral HM is consistent with previous data which indicated that renal PTH-like activity was also highest in patients without bone metastases (Ralston *et al.*, 1987) and may explain why the renal component of HM is unresponsive to bone-specific agents. The poor response to IVBPT in patients with humoral malignancy may reflect the high circulating levels of PTHrP in this group, and its effect in promoting renal reabsorption of calcium. A significant inverse relationship between plasma PTHrP 50-69 levels and the response to pamidronate as judged by the time taken to achieve normocalcaemia has been observed (Dodwell *et al.*, 1991), while elevated plasma PTHrP 53-84 was associated with a poor response to IVBPT in five out of six patients (Blind *et al.*, 1993). The biological effects of PTHrP on the renal tubule appear to be mediated via the PTH receptor, and a synthetic analogue, Tyr34 bPTH(7-34)$NH_2$, can inhibit the renal effects of PTH as a result of direct competition at the PTH-specific receptor site (Horiuchi *et al.*, 1983). Injection of this analogue in fetal lambs has been shown to inhibit the renal effects of PTHrP (Davicco *et al.*, 1992). Antibodies to PTHrP 1-34 have also been shown to lower serum calcium in a tumour model of hypercalcaemia in the athymic mouse (Kukreja *et al.*, 1988). Although it would be useful to be able to predict the effective dose or likely response of each patient to IVBPT, our data suggest that no single biochemical or clinical parameter is likely to be reliable. In the patients studied we found no correlation between plasma PTHrP and the magnitude of fall or nadir of serum calcium. Despite this, however, basal plasma PTHrP levels were higher in patients remaining hypercalcaemic. These data are the first to suggest that the response to therapy may reflect the aetiology of the hypercalcaemia, i.e. the presence or absence of bone metastases; patients with humoral hypercalcaemia and highest plasma PTHrP levels show the greatest resistance to therapy. Bisphosphonates will remain the standard treatment for hypercalcaemia associated with bone metastases; however, in future, treatment of humoral hypercalcaemia by bisphosphonates alone may be inappropriate. Combination therapy with competitive PTH analogues, or PTHrP monoclonal antibodies, may be beneficial to prevent the onset of humoral hypercalcaemia of malignancy, and to treat those in whom hypercalcaemia is refractory to current treatments.

### References

BLIND, E., RAUE, F., MEINEL, T., WUSTER, C. & ZIEGLER, R. (1993). Levels of parathyroid hormone related protein (PTHrP) in hypercalcaemia of malignancy are not lowered by treatment with the bisphosphonate BM.210955. *Horm. Metab. Res.*, **25**, 40-44.

BODY, J.J., BORKOWSKI, A., CLEEREN, A. & BIJVOET, O.L.M. (1986). Treatment of malignancy-associated hypercalcaemia with intravenous aminohydroxy-propilidene diphosphonate. *J. Clin. Oncol.*, **8**, 1177-1183.

BODY, J.J., DUMON, J.C., THIMON, M. & CLEEREN, A. (1993). Circulating PTHrP concentrations in tumour-induced hypercalcaemia: influence on the response to bisphosphonates and changes after therapy. *J. Bone Min. Res.*, **8**, 701-706.

BURTIS, W.J., BRADY, T.G., ORLOFF, J.J., ERSBAH, J.B., WARRELL, R.P., OLSON, B.R., WU, T.L., MITNICK, M.E., BROADUS, A.E. & STEWART, A.F. (1990). Immunochemical characterisation of circulating parathyroid hormone-related protein in patients with humoral hypercalcaemia of cancer. *N. Engl. J. Med.*, **322**, 1106-1112.

DANKS, J.A., EBELING, P.R. & HAYMAN, J. (1989). Parathyroid hormone-related protein: immunohistochemical localization in cancers and in normal skin. *J. Bone Min. Res.*, **4**, 273-278.

DAVICCO, M., COXAM, V., LEFAIVRE, J. & BARLET, J. (1992). Parathyroid hormone-related peptide increases urinary phosphate excretion in fetal lambs. *Exp. Physiol.*, **77**, 377-383.

DODWELL, D.J., ABBAS, S.K., MORTON, A.R. & HOWELL, A. (1991). Parathyroid hormone-related protein (50–69) and response to pamidronate therapy for tumour induced hypercalcaemia. *Eur. J. Cancer*, **12**, 1629–1633.

GALLACHER, S.J., FRASER, W.D., LOGUE, F.C., DRYBURGH, F.J., COWAN, R.A., BOYLE, I.T. & RALSTON, S.H. (1992). Factors predicting the acute effect of Pamidronate on serum calcium in hypercalcaemia of malignancy. *Calcif. Tissue*, **51**, 419–423.

GARDNER, M.D., DRYBURGH, F.J., FYFFE, J.A. & JENKINS, A.S. (1981). Predictive value of derived calcium figures based on the measurement of ionised calcium. *Ann. Clin. Biochem.*, **18**, 106–110.

GRILL, V., HO, P.W., BODY, J.J., JOHANSON, N., LEE, S.C., KUKREJA, S.C., MOSELEY, J.M. & MARTIN, T.J. (1991). Parathyroid hormone-related protein: elevated levels in both humoral hypercalcaemia of malignancy and hypercalcaemia complicating metastatic breast cancer. *J. Clin. Endocrinol. Metab.*, **73**, 1309–1315.

GRILL, V., MURRAY, M.L., HO, P.W.M., SANTAMARIA, J.D., PITT, P., POTTS, C., JERUMS, G. & MARTIN, T.J. (1992). Circulating PTH and PTHrP levels before and after treatment of tumour induced hypercalcaemia with pamidronate disodium (APD). *J. Clin. Endocrinol. Metab.*, **74**, 1408–1470.

GURNEY, H., GRILL, V. & MARTIN, T.J. (1993). Parathyroid hormone related-protein and response to pamidronate in tumour-induced hypercalcaemia. *Lancet*, **341**, 1611–1613.

HORIUCHI, N., HOLICK, M.F., POTTS, J.T. & ROSENBLATT, M. (1983). A parathyroid hormone inhibitor *in vivo*: design and biological evaluation of a hormone analog. *Science*, **220**, 1053–1055.

JUPPNER, H., ABOU-SAMRA, A., UNENOS, S., GU, W., POTTS, J.T. & SEGRE, G.V. (1988). The parathyroid hormone-like peptide associated with humoral hypercalcaemia of malignancy and parathyroid hormone bind to the same receptor on the plasma membrane of POS 17/7-8 cells. *J. Biol. Chem.*, **263**, 8557–9560.

KRAMER, S., REYNOLDS, F.H., CASTILLO, M., VALENZUELA, D.M., THORNLAY, M. & SORVILLO, J.M. (1991). Immunological identification and distribution of parathyroid hormone-like protein polypeptides in normal and malignant tissues. *Endocrinology*, **128**, 1927–1937.

KUKREJA, S.C., SHEVRIN, D.H., WIMBISCUS, S.A., EBELING, P.R., DANKS, J.A., RODDA, C.P., WOOD, W.I. & MARTIN, T.J. (1988). Antibodies to parathyroid hormone-related protein lower serum calcium in athymic mouse models of malignancy-associated hypercalcaemia due to human tumours. *J. Clin. Invest.*, **82**, 1798–1802.

MARTIN, T.J. & SUVA, L.J. (1989): Parathyroid hormone related-protein in hypercalcaemia of malignancy. *Clin. Endocrinol.*, **31**, 631–647.

MOSELEY, J.M., HAYMAN, J.A., DANKS, J.A., ALCORN, D., GRILL, V., SOUTHBY, J. & HORTON, M.A. (1991). Immunohistochemical detection of parathyroid hormone-related protein in human fetal epithelia. *J. Clin. Endocrinol. Metab.*, **73**, 478–484.

PEACOCK, M., ROBERTSON, W.G. & NORDIN, B.E.C. (1969). Relation between serum and urinary calcium with particular reference to parathyroid hormone. *Lancet*, **i**, 384–386.

RALSTON, S.H., GARNDER, M.D. & JENKINS, A.S. (1987). Malignancy-associated hypercalcaemia: relationship between mechanisms of hypercalcaemia and response to antihypercalcaemic therapy. *Bone Min.*, **2**, 227–242.

RALSTON, S.H., PATEL, U. & FRASER, W.D. (1989). Comparison of three intravenous bisphosphonates in cancer-associated hypercalcaemia. *Lancet*, **ii**, 1180–1182.

RATCLIFFE, W.A., NORBURY, S., HEATH, D.A. & RATCLIFFE, J.G. (1991). Development and validation of an immunoradiometric assay of parathyrin-related protein in unextracted plasma. *Clin. Chem.*, **37**, 678–685.

RATCLIFFE, W.A., HUTCHESSON, A.C.J., BUNDRED, N.J. & RATCLIFFE, J.G. (1992). Role of assays for parathyroid hormone-related protein in investigation of hypercalcaemia. *Lancet*, **339**, 164–167.

RIZZOLI, T.J., NAGODE, L.A., COUTO, C.G., HAMMER, A.S., CHEW, D.J., PETERSON, J.L., AYL, R.D., STEINMEYER, C.L. & CAPEN, C.C. (1989). High extracellular calcium increases the production of a parathyroid hormone like activity by cultured Leydig tumour cells associated with humoral hypercalcaemia. *J. Bone Min. Res.*, **4**, 839–844.

THIEBAUD, D., JAEGER, P. & BURCKHARDT, P. (1986). A single day treatment of tumour induced hypercalcaemia by intravenous APD. *J. Bone Min. Res.*, **1**, 555–562.